# Positional effects of head and/or neck flexion as chin-down posture in normal subjects

**DOI:** 10.20407/fmj.2019-018

**Published:** 2020-03-25

**Authors:** Megumi Ozeki, Hitoshi Kagaya, Yoko Inamoto, Tomoko Iizumi, Seiko Shibata, Keiko Onogi, Eiichi Saitoh

**Affiliations:** 1 Faculty of Rehabilitation, School of Health Sciences, Fujita Health University, Toyoake, Aichi, Japan; 2 Department of Rehabilitation Medicine I, School of Medicine, Fujita Health University, Toyoake, Aichi, Japan; 3 Department of Communication Disorders, School of Rehabilitation Science, Health Sciences University of Hokkaido, Hokkaido, Japan; 4 Faculty of Nursing, School of Health Sciences, Fujita Health University, Toyoake, Aichi, Japan

**Keywords:** Head flexion, Neck flexion, Combined head-and-neck flexion, Chin-down, Dysphagia

## Abstract

**Objective:**

The “chin-down” posture involves tucking the chin to the neck. However, clinicians and researchers have their own forms of the chin-down posture: some consider it to be head and neck flexion, whereas others consider it to be head flexion alone. The purpose of this study was to evaluate the effects of head, neck and combined head-and-neck flexion postures separately.

**Methods:**

Ten healthy volunteers participated in the study. The head and neck were set in neutral (N), head flexion (HF), neck flexion (NF) or combined head-and-neck flexion (HFNF) positions. Participants were instructed to swallow 4 ml of thick barium liquid in an upright sitting position. Head and neck angles at rest, distances in the pharynx and larynx at rest, and duration of swallowing were measured. Statistical analysis was performed with a paired t-test with Bonferroni correction.

**Results:**

Head angles in HF, NF and HFNF positions were significantly greater than in the N position. Neck angles were significantly greater in the NF position than in the N position. The distance between the tongue base and the posterior pharyngeal wall, the vallecular space and the airway entrance were smaller in the HF position than in the N position. The tongue base was in contact with the posterior pharyngeal wall longer in the HF position than in the N position.

**Conclusion:**

Because HF, NF and HFNF positions have different effects, we recommend the use of these terms instead of “chin-down position.”

## Introduction

Dysphagia hinders oral intake. Many compensatory postures have been advocated to treat dysphagia. The “chin-down” posture is one of the most frequently used in dysphagia rehabilitation, with many researchers commenting on its effects.^1–8^ Logemann^4^ suggested that the chin-down posture is helpful in patients with delayed triggering of the pharyngeal swallow, reduced tongue base retraction, and/or reduced airway entrance closure. In many patients, this posture pushes the tongue base and epiglottis closer to the posterior pharyngeal wall, narrows the airway entrance, and widens the vallecular space. However, other researchers have claimed that the chin-down posture narrows the vallecular space and does not affect the airway entrance.^3^

Although the chin-down posture involves tucking the chin to the neck, presumably via flexion of the head and/or neck, the posture is not clearly defined. Anatomically, head flexion arises from flexion of the atlanto-occipital and C1–C2 joints, whereas neck flexion refers to flexion in the lower cervical spine.^9^ A questionnaire survey showed that most speech-language pathologists in the United States recognize the chin-down posture to be a combination of head and neck flexion. In Japan, however, more than half of speech-language-hearing therapists consider the chin-down posture to be head flexion alone.^10^

Different definitions could result in inconsistency in the reported effects of the chin-down posture. The purpose of this study was to evaluate the effects of head, neck, and combined head-and-neck flexion postures separately.

## Methods

Ten healthy adults (seven men and three women; mean age 38 years) with no history of stroke or of oropharyngeal, neuromuscular, head or neck problems associated with swallowing participated in this study. Written informed consent was obtained from all participants. This study was approved by the ethical committee at our institution.

**Figure 1:**
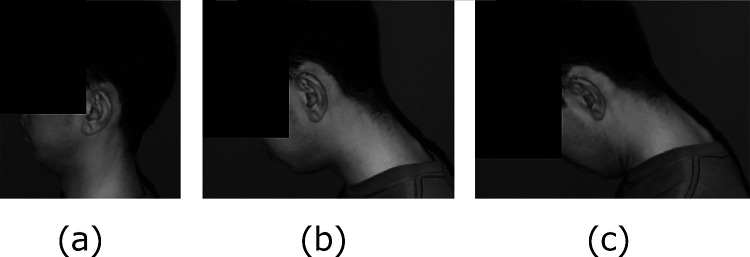
Head and neck positions (a) Head flexion. (b) Neck flexion. (c) Combined head-and-neck flexion. Head flexion arises from flexion of the atlanto-occipital and C1–C2 joints, whereas neck flexion indicates flexion in the lower cervical spine. Note that the chin is close to the neck in positions (a) and (c).

Head and neck positions were set at neutral (N), head flexion (HF), neck flexion (NF) or combined head and neck flexion (HFNF) (Figure 1). Participants were instructed to flex their head and/or neck to the maximum extent without pain and without causing swallowing difficulty. They were also instructed to maintain as stable a head and neck position as possible while swallowing. Participants were instructed to swallow 4 ml of barium liquid to which thickener (Thick and Easy; Hormel Health Labs, TX) had been added (4.5 g thickener in 100 ml of 50% w/v barium liquid). Participants swallowed while maintaining an upright sitting position.

Lateral views were recorded during videofluoroscopic examination of swallowing at a rate of 30 Hz. A 10.5-mm lead ball was positioned at the tip of the chin of each subject. The following parameters were evaluated.

### 1. Head angles at rest

**Figure 2:**
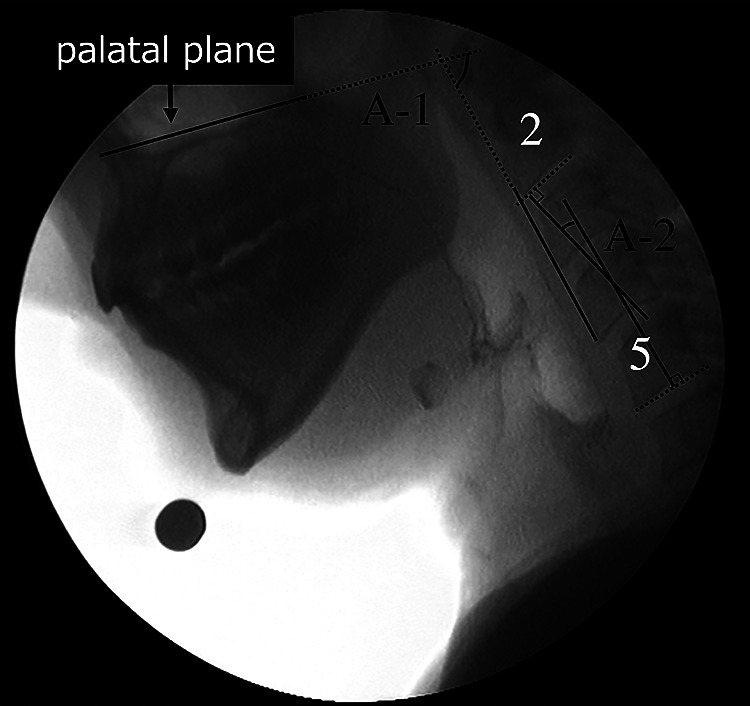
Head and neck angles A-1: Head angle The angle between the line connecting the anterior-inferior second and fourth cervical vertebrae and the palatal plane. A-2: Neck angle The angle between the line connecting the anterior and posterior aspects of the inferior second cervical vertebra and the line connecting the anterior and posterior aspects of the inferior fifth cervical vertebra.

The angle between the line connecting the anterior-inferior aspects of the second and fourth cervical vertebrae and the palatal plane (the line connecting the anterior and posterior nasal spine) (Figure 2, A-1).

### 2. Neck angles at rest

The angle between the line connecting the anterior and posterior aspects of the inferior second cervical vertebra and the line connecting the anterior and posterior aspects of the inferior fifth cervical vertebra (Figure 2, A-2).

### 3. Distances in the pharynx and larynx at rest

Three distances perpendicular to the line passing through the anterior-inferior aspects of the second and fourth cervical vertebrae were measured.

**Figure 3:**
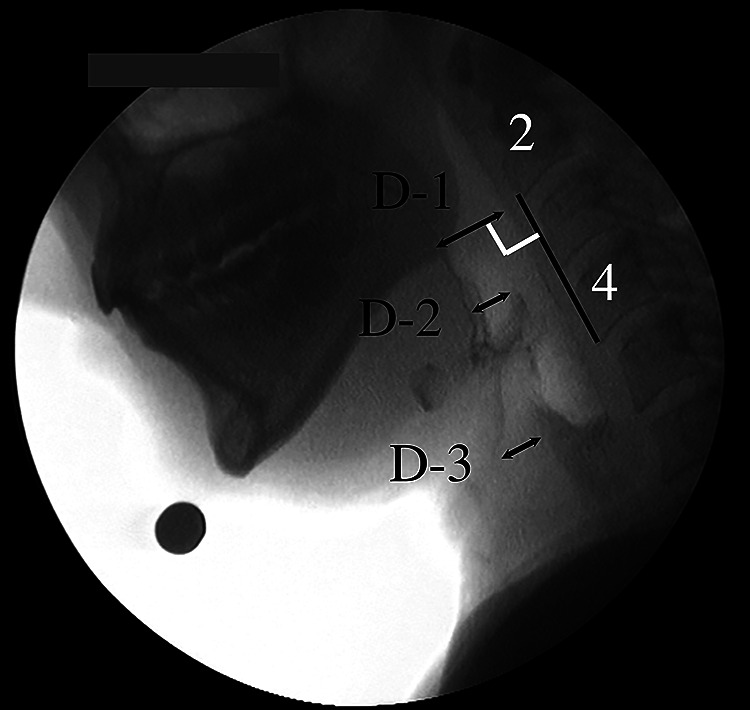
Distances perpendicular to the line passing through the anterior-inferior second and fourth cervical vertebrae. D-1: From the tongue base to the posterior pharyngeal wall D-2: From the uppermost anterior aspect of the epiglottis to the anterior pharyngeal wall (vallecular space) D-3: From the anterior-most surface of the arytenoid cartilage to the anterior wall of the laryngeal vestibule (airway entrance)

(a)  from the tongue base to the posterior pharyngeal wall (Figure 3, D-1)
(b)  from the uppermost anterior part of the epiglottis to the anterior pharyngeal wall (vallecular space) (Figure 3, D-2)
(c)  from the anterior-most surface of the arytenoid cartilage to the anterior wall of the laryngeal vestibule (airway entrance) (Figure 3, D-3)

### 4. Duration of swallowing

(a)  duration of contact between the tongue base and the posterior pharyngeal wall at the level of the inferior second cervical vertebra
(b)  duration of contact between the arytenoids and the epiglottis (laryngeal closure duration)
(c)  duration from the time at which the bolus passes the lower border of the ramus of the mandible to the time at which maximal excursion of the hyoid is initiated (stage transition duration)^11^
(d)  duration of opening of the upper esophageal sphincter (UES) 1 cm distal to the vocal folds (UES opening duration)^12^
(e)  maximum anteroposterior diameter of the UES 1 cm distal to the vocal folds (maximum UES opening width)^12^

### Statistical analysis

A paired t-test with Bonferroni correction was used to determine the significance of differences in the HF, NF and HFNF positions compared with the N position. P values of < 0.05/3 were considered statistically significant.

## Results

Head angles in the HF (P=0.002), NF (P=0.007) and HFNF (P<0.001) positions were significantly greater than in the N position. Neck angles were significantly larger in the NF position than in the N position (P=0.007). The distance from the tongue base to the posterior pharyngeal wall (P=0.002), the vallecular space (P<0.001) and the airway entrance (P<0.001) were smaller in the HF position than in the N position. In the HF position, the tongue base was in contact with the posterior pharyngeal wall longer than in the N position (P=0.013). No significant differences were found among positions in laryngeal closure duration, stage transition duration, UES opening duration or maximum UES opening width (Table 1). None of the participants in this study aspirated boluses in any of the trials.

## Discussion

We found that the head angle increased not only in HF, but also in NF and HFNF positions. However, only the HF position decreased the distance between the tongue base and the posterior pharyngeal wall, the vallecular space and the size of the airway entrance. The effects of the chin-down posture on the vallecular space and the airway entrance are controversial.^2–5^ We suggest that disagreements derive from inconsistencies in the definition of “chin-down” posture and that only the HF position narrows the vallecular space and the airway entrance. Because the chin-down posture is reported to enhance closure of the laryngeal vestibule,^1^ HF may be useful for patients with incomplete laryngeal closure.

We also found that the tongue base was in contact with the posterior pharyngeal wall longer in the HF position than in the N position. Pharyngeal pressure increases when the tongue base is in contact with the posterior pharyngeal wall.^13,14^ Therefore, HF may be effective in patients with reduced pharyngeal constriction and with reduced tongue base retraction. Neck angles increased significantly in the NF position only. An increased neck angle prevents the bolus from dropping directly from the pharynx to the trachea. Because the participants in this study had no swallowing problems, nobody aspirated boluses in any position. We need to confirm these results in patients with dysphagia.

Logemann^4^ suggested that the chin-down posture may be helpful in patients with delayed triggering of the pharyngeal swallow because material is more likely to remain in the valleculae long enough to trigger a pharyngeal swallow. A prolonged duration of stage transition delays the swallowing reflex^11^; however, posture did not affect the duration of stage transition in this study. We hypothesize that the chin-down posture does not delay the swallow onset, at least in normal individuals. Ohmae et al.^12^ reported that the maximum UES opening width increased during super-supraglottic swallowing in healthy young men. In contrast, our study did not show differences in UES opening time or in maximum UES opening width among different postures, indicating that the chin-down posture does not affect the UES.

Our study has some limitations. The alignment of the cervical spine differs among individuals, so the effects of HF, NF and HFNF must be variable. We did not evaluate the effect of these positions in patients with dysphagia. None of the participants in this study aspirated boluses; however, Ra et al.^15^ reported that a chin-down posture reduced or eliminated aspiration in 19.6% of patients. Further evaluation of the effects of HF, NF and HFNF positions in patients with dysphagia is necessary in a future study.

## Conclusions

The term “chin-down” means tucking the chin to the neck. Clinicians and researchers appear to have their own “chin-down” postures, which can lead to confusion. In this study, we observed that the HF position pushed the tongue base and epiglottis close to the posterior pharyngeal wall at rest and resulted in a longer duration of contact between the tongue base and the posterior pharyngeal wall during swallowing. In addition, the vallecular space and the airway entrance were narrowed in the HF position. We recommend the use of the terms “head flexion,” “neck flexion” and “head-and-neck flexion” instead of “chin-down posture.” We believe that the correct use of these technical terms will contribute to scientific progress by removing possible confusion. The effects of these positions in patients with dysphagia should be evaluated in the near future to further our understanding of their possible benefits.

## Figures and Tables

**Table 1:**
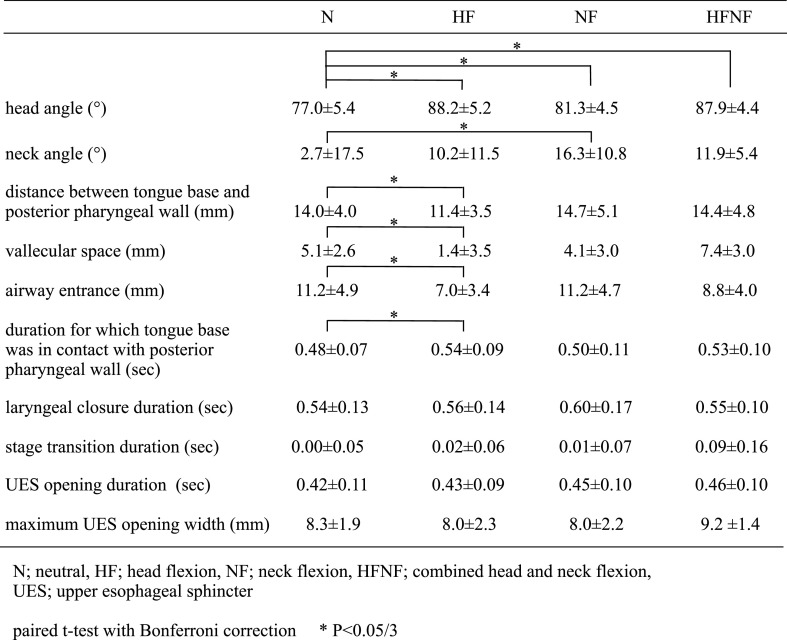
Positional effects of N, HF, NF, and HFNF
